# Ameliorative Effects of Antioxidants on the Hippocampal Accumulation of Pathologic Tau in a Rat Model of Blast-Induced Traumatic Brain Injury

**DOI:** 10.1155/2016/4159357

**Published:** 2015-12-16

**Authors:** Xiaoping Du, Matthew B. West, Weihua Cheng, Donald L. Ewert, Wei Li, Debra Saunders, Rheal A. Towner, Robert A. Floyd, Richard D. Kopke

**Affiliations:** ^1^Hough Ear Institute, Oklahoma City, OK 73112, USA; ^2^Oklahoma Medical Research Foundation, Oklahoma City, OK 73104, USA; ^3^Departments of Physiology and Otolaryngology, University of Oklahoma Health Sciences Center, Oklahoma City, OK 73104, USA

## Abstract

Traumatic brain injury (TBI) can lead to early onset dementia and other related neurodegenerative diseases. We previously demonstrated that damage to the central auditory pathway resulting from blast-induced TBI (bTBI) could be significantly attenuated by a combinatorial antioxidant treatment regimen. In the current study, we examined the localization patterns of normal Tau and the potential blast-induced accumulation of neurotoxic variants of this microtubule-associated protein that are believed to potentiate the neurodegenerative effects associated with synaptic dysfunction in the hippocampus following three successive blast overpressure exposures in nontransgenic rats. We observed a marked increase in the number of both hyperphosphorylated and oligomeric Tau-positive hilar mossy cells and somatic accumulation of endogenous Tau in oligodendrocytes in the hippocampus. Remarkably, a combinatorial regimen of 2,4-disulfonyl *α*-phenyl tertiary butyl nitrone (HPN-07) and *N*-acetylcysteine (NAC) resulted in striking reductions in the numbers of both mossy cells and oligodendrocytes positively labeled for these pathological Tau immunoreactivity patterns in response to bTBI. This treatment strategy represents a promising therapeutic approach for simultaneously reducing or eliminating both primary auditory injury and nonauditory changes associated with bTBI-induced hippocampal neurodegeneration.

## 1. Introduction

Traumatic brain injury (TBI) is a serious clinical challenge that negatively impacts millions of people worldwide. Repetitious low-impact concussive events or a single severe TBI can lead to early onset dementia and other related neurodegenerative diseases. Blast-induced TBI (bTBI) is a common form of TBI suffered in both military and civilian populations. Axonal injury is one of the common sequelae of bTBI [1–5]. Diffuse axonal injuries have been detected in most regions of the brain, including the cortex and white matter, the hippocampus and medial geniculate nucleus, and the cerebellum and brainstem following blast exposure [1–8]. This type of diffuse axonal injury can result directly from blast-induced mechanical shearing, resulting in discontinuities in neurotransmission [9]. Alternatively, and often in parallel with direct mechanical damage, bTBIs can induce maladaptive molecular cascades that lead to progressive waves of ongoing neurodegeneration [10]. This progressive, posttraumatic pathophysiological response has become a hallmark of bTBI and other clinically related neuropathies associated with concussive brain traumas, such as chronic traumatic encephalopathy (CTE).

Like CTE, the ongoing neurodegeneration associated with bTBIs has been shown to be a Tau protein-linked disorder, or tauopathy [10, 11]. In uninjured brains, the soluble microtubule-associated protein, Tau, is enriched in neuronal axons where it plays a key role in stimulating microtubule formation, outgrowth, and subsequent maintenance of cytoskeletal stability, thus promoting axonal and dendritic transport [12]. However, Tau is susceptible to stress-induced hyperphosphorylation in response to single or repetitive neurotraumas. Hyperphosphorylated Tau is prone to misfolding and aggregation, leading to destabilization of microtubules and, thus, compromised neuronal viability and function. Moreover, once initiated, stress-induced destabilization of Tau can result in propagative waves of Tau dysfunction, as hyperphosphorylated Tau inclusions have a propensity to recruit and subvert functional Tau proteins in a prion-like manner that can progress in a transcellular fashion [13, 14]. Although the neurofibrillary tangles (NFTs) that result from this pathophysiological process have long been appreciated as a central feature of neuropathies, such as Alzheimer's disease and dementia, it is now recognized that the oligomeric “seeds” of hyperphosphorylated Tau that initiate the formation of NFTs are sufficient for the neuronal cell death and cognitive deficits associated with these disorders [15]. Thus, early intervention strategies aimed at attenuating or eliminating the etiological precursors of NFTs would have significant clinical impact on both the acute and chronic manifestations of bTBI-related cognitive dysfunction.

While the precise mechanisms of Tau hyperphosphorylation and oligomerization associated with bTBIs remain unclear, oxidative stress is likely to be a key pathological component in this process. It has been demonstrated both* in vitro* and* in vivo* that aberrant Tau phosphorylation is potentiated by oxidative stress and is a reflection of the extent to which neuronal cells can combat free radical-induced oxidative and nitrosative damage resulting from neurodegenerative mitochondrial dysfunction [16]. Moreover, mild TBIs resulting from blast overpressures have been shown to induce prolonged oxidative stress and concomitant increases in antioxidant enzyme activity, especially in regions of the brain that are particularly susceptible to acute and chronic manifestations of bTBI, such as the hippocampus [4, 7, 17–23]. As a result, therapeutic intervention strategies designed to ameliorate both acute stress and sustained oxidative stress following a bTBI or other related tauopathies may short-circuit the underlying physiological machinery that promote progressive neurodegeneration.

Despite these observations, there is currently no effective treatment strategy for addressing the cognitive dysfunction and adverse physiological responses associated with TBI. Using a nontransgenic rat model, our laboratory previously demonstrated that damage to the central auditory pathway resulting from mild successive bTBIs, a common form of neurotrauma encountered in battlefield conditions, could be significantly attenuated by a combinatorial antioxidant treatment regimen of* N*-acetylcysteine (NAC) and 2,4-disulfonyl *α*-phenyl tertiary butyl nitrone (HPN-07) following exposure to blast overpressures which resulted in pervasive oxidative stress and sustained neurodegeneration in the brains of untreated rats [7, 24]. In the present study, we demonstrate that this treatment strategy is also capable of reducing bTBI-induced accumulation of neuropathological forms of Tau in the hippocampus, a primary center in the brain associated with blast-induced pathophysiological responses.

## 2. Methods and Materials

### 2.1. Animals, Blast Exposure

This study was carried out in strict accordance with the recommendations in the Guide for the Care and Use of Laboratory Animals of the National Institutes of Health. All animal procedures were approved by the Oklahoma Medical Research Foundation (OMRF) Institutional Animal Care and Use Committee (Permit Number: K0133-1) and the US Department of the Navy Office of Naval Research (Permit Number: NRD-561). Male Long-Evans pigmented rats with body weights between 360 and 400 g were purchased from Harlan Laboratories (Indianapolis, Indiana). The animals were housed and maintained in the animal care facility at OMRF.

The blast exposure paradigm and administration of antioxidant treatments have been detailed previously [24]. In brief, blasts were generated by a custom-built blast simulator using compressed nitrogen against a plastic film. The top of the rat head was positioned perpendicular to the nozzle of the blast simulator, while its body was protected by a holding tube. Each rat was exposed to 14 psi blasts repeated three times at 1.5-minute intervals under deep anesthesia (50 mg/kg of ketamine and 6 mg/kg of xylazine). This blast exposure paradigm consistently produced significant permanent hearing loss with a low incidence of tympanic membrane rupture and significant brain injury in multiple regions including the hippocampus [7, 24].

### 2.2. Grouping and Drug Administration

After blast exposure, rats were randomly assigned to either an antioxidant treatment group (blast plus treatment, B/T), which received 300 mg/kg NAC (Hospira, Inc., Lake Forest, IL) plus 300 mg/kg HPN-07 (APAC Pharmaceuticals LLC, Columbia, MD), or a blast control group (B), which received an equivalent volume of saline only. A combination of NAC and HPN-07 was used in the current study due to the documented synergistic efficacy of these drugs in ameliorating the damaging effects of noise- and blast-induced traumas on both progressive hearing loss and brain injury [7, 24–26]. Each group included 16 to 24 rats. NAC and HPN-07 were dissolved together in a physiological saline solution (60 mg/mL for each drug) and were intraperitoneally injected into animals in the treatment group, beginning one hour after blast exposure and then twice daily for the following two days. An equal volume of physiological saline solution was injected into rats in the blast control group, according to the same schedule as that maintained for the treatment group. An additional six rats that were neither exposed to blast nor received drug treatments were used as normal controls (NCs). For the data presented in the current study, animals in each group (6–8 rats/time point) were euthanized and intracardially perfused with 4% paraformaldehyde in 0.1 M phosphate-buffered saline (pH 7.2) at either 24H, 7D, or 21D after blast.

### 2.3. Collection and Sectioning of Brains

All tissue samples were collected and prepared for histological examination as described previously [7, 24]. In brief, brains were removed and postfixed in the same fixative for one week and then washed and stored in PBS at 4°C. The brain from each animal was cryoprotected in 30% sucrose in PBS until the tissue had settled to the bottom of the container. Cryoprotected tissues were then embedded in Tissue-Tek (Sakura Finetek USA Inc., Torrance, CA) and serially sectioned in a coronal plane with a Thermo Cryotome (Thermo Fisher Scientific, Inc., Waltham, MA) at 18–20 *μ*m. Every tenth section from each brain was mounted onto a gelatin precoated slide (total of 20 slides for each brain, with 10–12 sections on each slide).

### 2.4. Immunohistological Analyses

The brain sections were washed with PBS, blocked in 1% bovine serum albumin (fraction V) and either 1% normal horse serum or 1% normal goat serum in PBS, and permeabilized in 0.2% Triton X-100 in PBS (PBS/T). The tissues were then incubated overnight with either mouse anti-Tau-1 antibody, which recognizes all known electrophoretic species of Tau protein lacking phosphorylation at serine sites 195, 198, 199, and 202 [1 : 200, clone PC1C6, EMD Millipore, Billerica, MA], mouse anti-phospho-PHF-Tau pSer202/Thr205 antibody (1 : 250, clone AT8, Thermo Scientific), or rabbit anti-oligomeric Tau antibody (T22 serum, 1 : 300, a kind gift from Dr. Rakez Kayed at the University of Texas Medical Branch, Galveston, TX). After washing with PBS/T, either biotinylated anti-rabbit IgG or anti-mouse IgG (1 : 200, Vector Laboratories, Inc., Burlingame, CA) was applied to the slides for one hour, and Vectastain ABC and DAB kits (Vector Laboratories, Inc., Burlingame, CA) were used for the immunolabeling visualization. Immunopositive cells exhibited a brown reaction product at the sites of the target epitopes. Methyl green was used for nuclear counterstaining.

To identify the cell types that differentially accumulated hyperphosphorylated or oligomeric Tau, brain sections were coincubated with mouse anti-Tau-1 and either rabbit oligodendrocyte specific protein antibody (OSPA, 1 : 100, Cambridge, MA), rabbit anti-calretinin IgG (1 : 500, EMD Millipore, Billerica, MA), or rabbit anti-GluR 2/3 IgG (1 : 50, EMD Millipore, Billerica, MA); with mouse anti-AT8 IgG and either rabbit anti-calretinin IgG or rabbit anti-GluR 2/3 IgG; and with mouse anti-AT8 IgG and rabbit anti-T22 serum. After washing with PBS, the sections were then incubated with appropriate Alexa Fluor 488 or 568 secondary antibodies (1 : 1000, Life technologies, Co., Grand Island, NY). DAPI (4′,6-diamidino-2-phenylindole) was used to label nuclei. Fluorescence labeling was examined with either an Olympus BX51 microscope (Melville, NY) or a Zeiss LSM-710 confocal microscope (Carl Zeiss Microimaging, LLC, NY). Negative controls were prepared for each staining by omitting the primary antibody incubation step to control for specificity of immunolabeling.

### 2.5. Quantification of Pathologic Tau Immunostaining

For quantification of immunostaining, images were collected from the polymorphic layer (PoDG) and the infrapyramidal blade of the granular cell layer of the dentate gyrus (GrDG) of 5-6 sections of each brain. A modified two-dimensional quantification method was employed to count positive immunostained cells in these regions [7, 25]. Images were collected with a color camera (DP70) attached to an Olympus microscope (BX51) and DPController and DPManager programs (Olympus, Melville, NY). The distance between two adjacent sections on each slide was about 200 *μ*m to ensure nonduplicate counting. The total number of positive cells within each area was counted using Image J software (National Institutes of Health), and cell density was calculated (cells/mm^2^) and statistically analyzed as detailed below. Only cells with dark brown cytoplasmic staining (Tau-1 and AT8) or both cytoplasmic and nuclear staining (T22) were counted. Cell counting was blindly conducted by a technician who was unaware of the identity of each slide.

### 2.6. Statistical Analyses

All parameters measured are expressed as means ± standard error of the mean (SEM). One-way ANOVA (SPSS 14.0 for windows) was used to determine if there were statistically significant differences among the three experimental groups (NC, B, and B/T) at each time point. When a significant difference among groups was found, a post hoc test (Tukey HSD) was used to determine if there were statistically significant differences between group pairings (i.e., NC versus B; NC versus B/T; B versus B/T at each time point). Statistical analyses were conducted using GraphPad Prism 4 software (GraphPad Software, Inc., La Jolla, CA). A *p* value of less than 0.05 was considered to be significant.

## 3. Results

### 3.1. Effects of Blast Exposure and Antioxidant Treatment on Somatic Tau Staining in the Hippocampus

**Figure 1:**
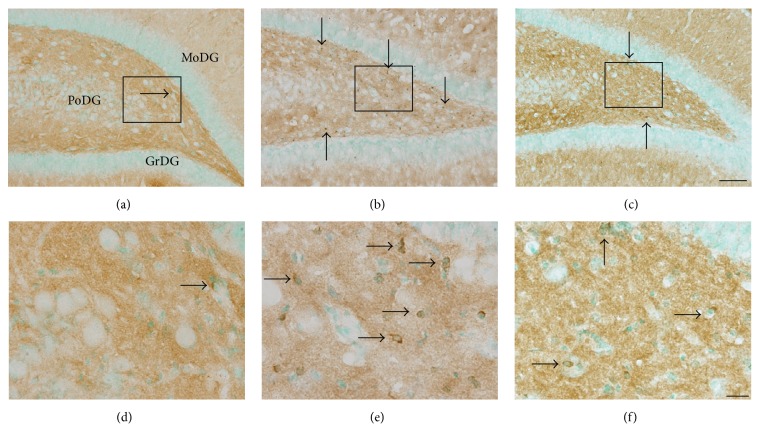
Antioxidant treatment reduces the blast-induced accumulation of somatic Tau in the hippocampus. Representative images of normal Tau (Tau-1) staining in the polymorphic layer of the dentate gyrus (PoDG) of the hippocampus from normal controls ((a) and (d)) and from untreated ((b) and (e)) or antioxidant-treated ((c) and (f)) rats seven days following blast trauma. The rectangles in (a)–(c) indicate the locations from which images were collected for (d)–(f), respectively. Cells positive for somatic Tau-1 immunostaining were observed in the hippocampus of all three groups, albeit rarely in normal controls (arrows in (a)–(f)). Scale bars = 200 *μ*m in (c) for (a)–(c) and = 20 *μ*m in (f) for (d)–(f).* GrDG*, granular layer of the dentate gyrus;* MoDG*, molecular layer of the dentate gyrus.

**Figure 2:**
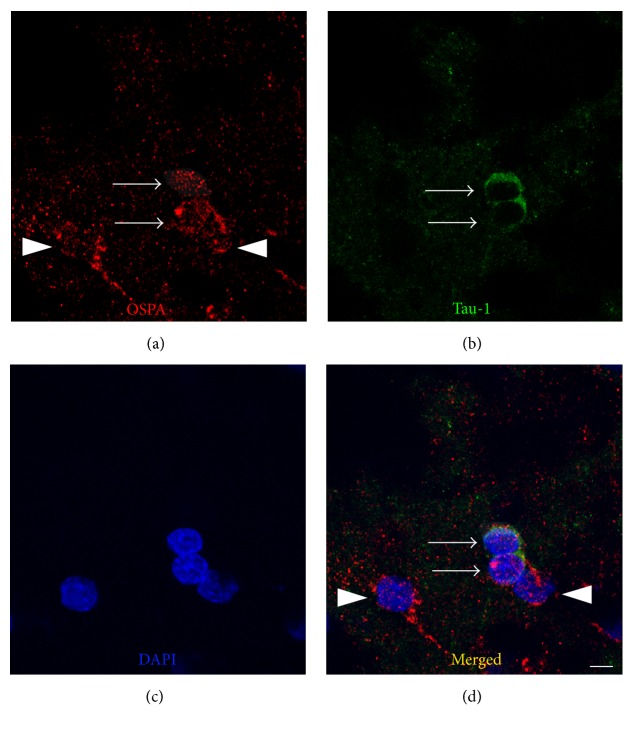
Tau accumulates in the soma of hippocampal cells in response to bTBI seven days after blast exposure. Some Tau-1-positive cells (arrows in (b)) exhibited colabeling with the oligodendrocyte-specific protein antibody (OSPA, arrows in (a) and (d)) in blast-exposed rats, while some oligodendrocytes were not colabeled with the Tau-1 antibody (arrowheads in (a) and (d)). The scale bar in (d) = 5 *μ*m for (a)–(d).

**Figure 3:**
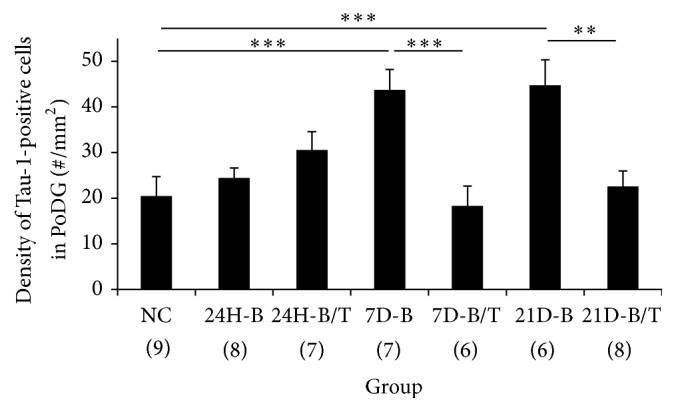
Quantification of somatic Tau-1 immunostaining in the hippocampus. Number of cells with Tau-1-positive somatic staining in the PoDG of the hippocampus in normal control rats (NC), rats exposed to blast (B), or rats exposed to blast followed by antioxidant treatment (B/T) were quantified and statistically analyzed. While there were no significant differences in the localization pattern of Tau-1 in the hippocampus 24 hours after blast exposure (all *p* > 0.05), a significantly increased number of Tau-1-positive somata were observed in the PoDG of the hippocampus at both seven and 21 days after blast compared to the normal controls (*p* < 0.001). Antioxidant treatment significantly reduced the somatic accumulation of Tau-1 in the PoDG of the hippocampus compared to untreated blast-exposed controls at these time points after exposure (*p* < 0.01 or < 0.001). ^*∗∗*^ *p* < 0.01 and ^*∗∗∗*^ *p* < 0.001. Error bars represent standard error of the means.

In uninjured brains, Tau localization is restricted to axons, resulting in a diffuse immunoreactivity pattern throughout the cortex and subcortical regions (Figures 1(a) and 1(d)) [27]. However, using an antibody against normal Tau protein (Tau-1), we observed a blast-induced accumulation of Tau-1-positive foci in the polymorphic layer of the dentate gyrus (PoDG) seven days after injury (Figures 1(b) and 1(e)). Upon closer examination, this atypical Tau immunostaining pattern was localized to the soma of small round cells, bearing large nuclei with limited axonal projections (arrows in Figures 1(d)–1(f)). These morphological features and distinct trauma-induced somatic Tau accumulation are consistent with characteristics associated with oligodendrocytes [28–33]. To confirm this characterization, relevant tissue sections were immunolabeled with oligodendrocyte specific protein antibody (OSPA) for colocalization evaluations with Tau-1. From this analysis, we determined that a large proportion of the Tau-1-positive cells were also OSPA-positive, confirming their characterization as oligodendrocytes, an example of which is shown in Figure 2. Formal quantification of these Tau-positive cell bodies in the PoDG revealed that bTBI induced a significant, yet delayed, increase in aberrant Tau-1 immunoreactivity in this region of the hippocampus, first evident at seven days after injury and persisting through the terminal sampling time point of 21 days after bTBI (*p* < 0.001, Figure 3).

We previously demonstrated that a combinatorial treatment regimen with the antioxidants HPN-07 and NAC could attenuate blast-induced neurodegeneration in several regions of the brain, including the hippocampus [7]. To assess whether these therapeutic effects extended to the blast-induced mislocalization of Tau-1 in hippocampal oligodendrocytes, the brain tissues from rats subjected to the same blast exposure paradigm followed by antioxidant intervention were examined for Tau-1 immunoreactivity. Remarkably, blast-exposed animals treated with a combinatorial regimen of the antioxidants HPN-07 and NAC exhibited striking reductions in somatic Tau-1 immunoreactivity in the hippocampus, such that, at both the seven- and 21-day time points after bTBI (*p* < 0.01 or 0.001, Figure 3), the number of atypical Tau-1-positive foci in this region of the brain was statistically indistinguishable from uninjured controls (*p* > 0.05, Figure 3). Taken together, these results indicate that the blast-induced somatic accumulation of Tau in oligodendrocytes within the hippocampus is, at least in part, governed by oxidative stress and antioxidant treatment can reduce this accumulation.

### 3.2. Effects of Blast Exposure and Antioxidant Treatment on the Accumulation of Hyperphosphorylated Tau in the Hippocampus

**Figure 4:**
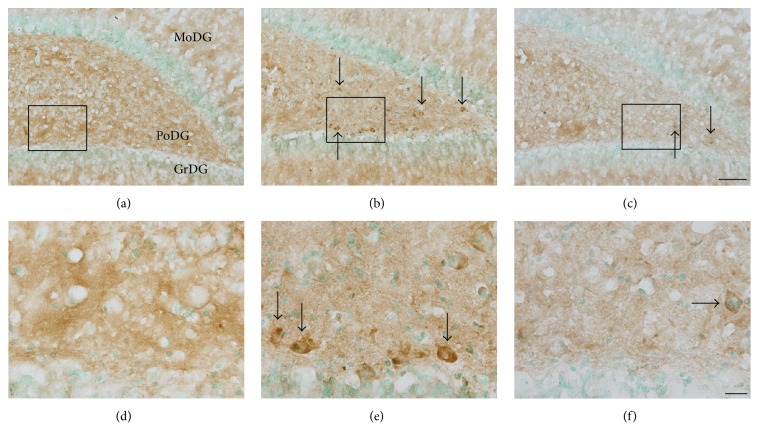
Antioxidant intervention opposes blast-induced hyperphosphorylation of Tau in the hippocampus. Representative images of hyperphosphorylated Tau (AT8) immunostaining in the dentate gyrus of the hippocampus from normal controls ((a), (d)), blast-exposed rats seven days after injury ((b), (e)), and antioxidant-treated rats seven days after blast ((c), (f)). The rectangles in (a)–(c) indicate the locations from which images were collected for (d)–(f), respectively. AT8-positive cells are denoted by arrows in (b)-(c) and (e)-(f). Scale bars = 200 *μ*m in (c) for (a)–(c) and = 20 *μ*m in (f) for (d)–(f).

Dysregulation of Tau phosphorylation can lead to progressive pathological processes that culminate in neuronal cell death [34]. To discern whether our blast model resulted in this type of etiopathogenic Tau modification, we probed brain tissues from blast-exposed rats with an antibody (AT8) that recognizes a hyperphosphorylated (Ser202, Thr205) Tau variant predisposed to form the paired helical filament (PHF) structures that comprise neurofibrillary tangles observed in CTE and related tauopathies [15]. At seven days after blast, many AT8-positive foci were observed throughout the PoDG (Figures 4(b) and 4(e)), a staining pattern which was comparatively absent in uninjured animals (Figures 4(a) and 4(d)). This pattern of blast-induced hyperphosphorylation of Tau was primarily localized within cells with relatively large somata that were morphologically distinct from the oligodendrocytes in which blast-induced Tau-1 mislocalization was observed at this same sampling interval (Figures 4(e) and 1(e)). However, as was observed for somatic Tau-1 accumulation in oligodendrocytes, the prevalence of these AT8-positive foci was reduced relative to untreated controls in blast-exposed animals treated with the combinatorial regimen of HPN-07 and NAC, indicative of a therapeutic effect of these antioxidants on the accumulation of this pathogenic Tau variant in this region of the hippocampus (Figures 4(c) and 4(f)).

**Figure 5:**
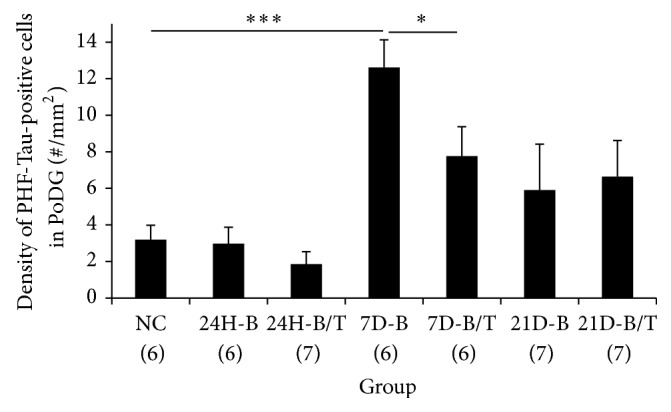
Quantification of hyperphosphorylated Tau accumulation in the hippocampus. Hyperphosphorylated Tau cell densities in the PoDG of the hippocampus from normal controls, blast-exposed rats, and blast-exposed rats subsequently treated with the antioxidants HPN-07 and NAC were calculated and statistically analyzed. Significant accumulation of hyperphosphorylated Tau was observed in the PoDG of the hippocampus seven days after blast exposure compared to uninjured controls (^*∗∗∗*^ *p* < 0.001). Significantly lower AT8-positive cell densities were observed in the PoDG of the hippocampus in blast-exposed animals subsequently treated with antioxidants (^*∗*^ *p* < 0.05) at this time point. No significant differences between experimental cohorts were observed at other time points (24H or 21D, all *p* > 0.05). However, the number of AT8-positive cells exhibited a significant decline between the seven- and twenty-one-day time points in blast-exposed rats (7D-B versus 21D-B, *p* < 0.05), while a similar time-dependent decline was not evident in blast-exposed rats subsequently treated with the antioxidants HPN-07 and NAC (7D-B/T versus 21D-B/T, *p* > 0.05). ^*∗*^ *p* < 0.05 and ^*∗∗∗*^ *p* < 0.001. Error bars represent standard error of the means.

To confirm these observations, AT8-positive somata in the PoDG were quantified, and cell densities were calculated and statistically analyzed. While no statistical differences in the number of AT8-positive somata were observed at 24 hours after blast (*p* > 0.05, Figure 5), a significant increase in hyperphosphorylated Tau was evident in the PoDG of the hippocampus seven days after blast exposure (*p* < 0.001, Figure 5). The apparent treatment effect of HPN-07 and NAC on the number of AT8-positive cells observed in the hippocampus at this time point after blast exposure was statistically confirmed, such that approximately 38.5% fewer cells exhibited pathologic Tau accumulation in antioxidant-treated animals than in untreated controls (*p* < 0.05, Figure 5). In contrast to the persistent somatic Tau-1 mislocalization observed in oligodendrocytes following blast, there were no significant differences in the number of AT8-positive cells in this region of the hippocampus at the terminal, 21-day time point after blast exposure relative to unexposed controls (all *p* > 0.05, Figure 5), perhaps indicating that this aberrant Tau phosphorylation pattern in the PoDG was resolved over time or that a significant proportion of the affected cells experienced neurotoxic attrition.

### 3.3. Effects of Blast Exposure and Antioxidant Treatment on Oligomeric Tau Staining in the Hippocampus

**Figure 6:**
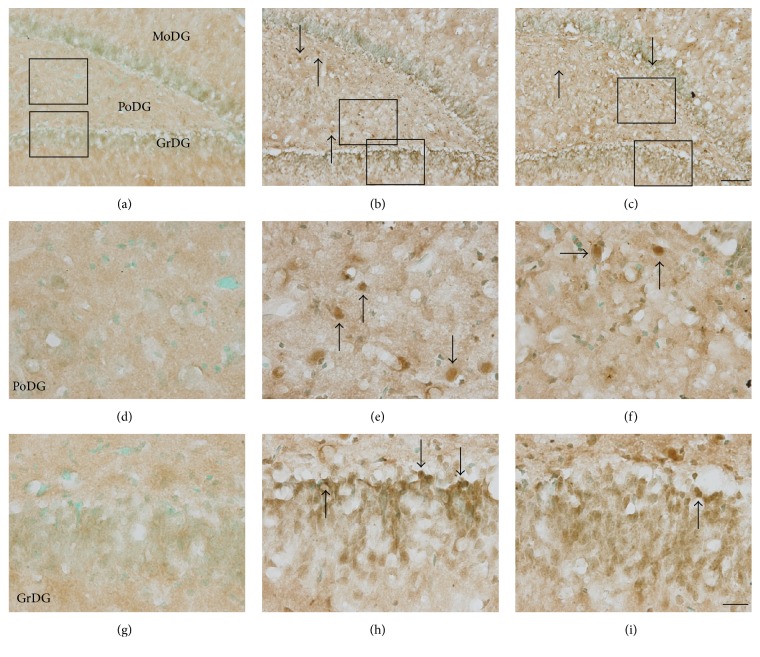
The blast-induced accumulation of pathologic Tau oligomers in the hippocampus is reduced by antioxidant intervention. Representative images of oligomeric Tau (T22) immunostaining in the dentate gyrus of the hippocampus from normal controls ((a), (d), and (g)), in blast-exposed rats seven days after injury ((b), (e), and (h)) and in antioxidant-treated rats seven days after blast ((c), (f), and (i)). The rectangles in (a)–(c) indicate the locations from which images were collected for (d)–(i). T22-positive cells are denoted by arrows. Scale bars = 200 *μ*m in (c) for (a)–(c) and = 20 *μ*m in (i) for (d)–(i).

**Figure 7:**
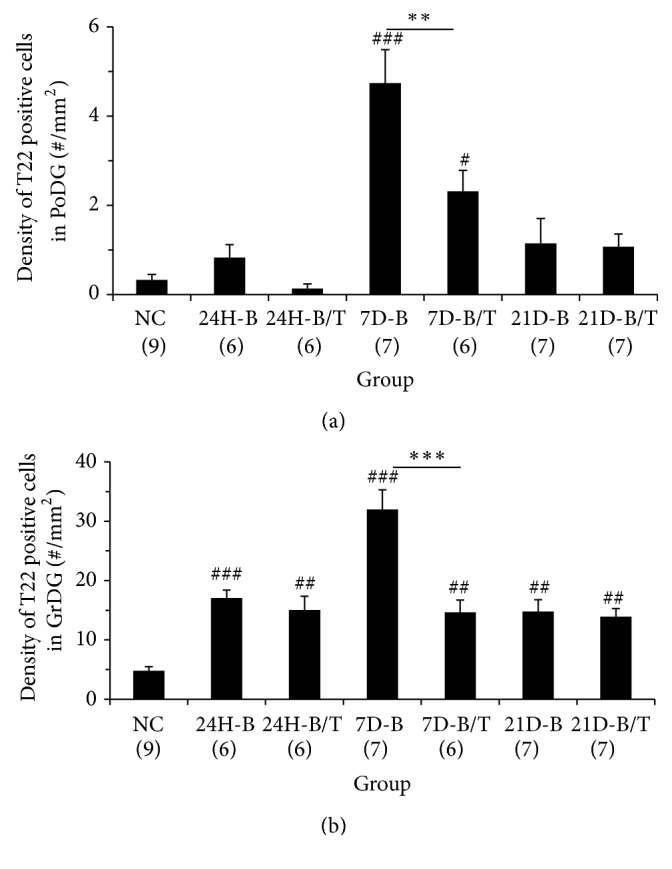
Quantification of blast-induced oligomeric Tau accumulation in the hippocampus. Densities of T22-positive cells in the PoDG (a) and GrDG (b) of the hippocampus from normal controls (NC), blast-exposed rats (B), and blast-exposed rats treated with a combinatorial antioxidant regimen (B/T) were calculated and statistically analyzed. In the PoDG, significantly more T22-positive cells were observed seven days after blast exposure in untreated rats (7D-B) compared to normal controls (*p* < 0.001). Antioxidant treatment significantly reduced the number of T22-positive cells in the PoDG seven days after blast exposure (7D-B/T, *p* < 0.01). There was a significant difference between NC and 7D-B/T groups (*p* < 0.05). In the GrDG, significantly more T22-positive cells were observed twenty-four hours to twenty-one days after blast exposure in untreated and treated rats compared to NCs although many more positive cells were observed at seven days in untreated rats (*p* < 0.01 or 0.001). Antioxidant treatment significantly reduced the number of T22-positive cells in the GrDG seven days after blast exposure (7D-B/T, *p* < 0.001). The number of T22-positive cells in both the PoDG and GrDG exhibited a significant decline between the seven- and twenty-one-day time points in untreated, blast-exposed rats (7D-B versus 21D-B, *p* < 0.001), a trend which was not observed in blast-exposed rats subsequently treated with the antioxidants HPN-07 and NAC (7D-B/T versus 21D-B/T, *p* > 0.05), likely owing to the marked treatment effect observed at the seven-day evaluation interval. These results indicate treatment effects of antioxidants on oligomeric Tau accumulation in the hippocampus. ^#^ *p* < 0.05, ^##^ *p* < 0.01, and ^###^ *p* < 0.001, compared to NC. ^*∗∗∗*^ *p* < 0.001. Error bars represent standard error of the means.

The formation of neurotoxic Tau oligomers precedes the establishment of neurofibrillary tangles, and their accumulation has been reported as an early diagnostic marker for traumatic brain injury [35, 36]. Using an antibody that specifically recognizes oligomeric Tau (T22) [36–38], we probed blast-exposed brain tissues to discern whether the appearance of these neurotoxic aggregates coincided with the other aberrant Tau immunoreactivity patterns observed in our model of bTBI. Indeed, a marked accumulation of T22 reactivity was observed within the dentate gyrus (granular cell and polymorphic layers) of the hippocampus seven days after blast exposure (Figure 6(b)). Strong somatic immunoreactivity was a common feature among these T22-positive cells (Figures 6(e) and 6(h)), a localization pattern which is consistent with that previously documented in a published study of rats exposed to fluid percussive TBIs [36]. The T22-positive cells observed in the PoDG (Figure 6(e)) were morphologically distinct from the Tau-1-positive oligodendrocytes (Figure 1(e)), more closely resembling the hippocampal cells in which AT8 immunoreactivity accumulated (Figure 4(e)). In the granular cell layer of the dentate gyrus (GrDG), T22-positive cells primarily accumulated along the infrapyramidal blade (Figures 6(b) and 6(h)). T22-positive cells in both the PoDG and GrDG of the hippocampus of blast-exposed rats were quantified, and their relative densities were calculated and statistically compared to the corresponding regions in untreated rats. Significantly greater numbers of T22-positive cells were observed in both of these hippocampal cellular layers of blast-exposed animals, first evident at 24 hours in the GrDG and at seven days in the PoDG after injury (all *p* < 0.001, Figures 7(a) and 7(b)). Although blast-induced somatic accumulation of both unphosphorylated (Tau-1-positive) and hyperphosphorylated (AT8-positive) Tau isoforms were largely restricted to the PoDG, greater numbers of T22-positive cells were detected in the GrDG than in the PoDG (Figures 7(a) and 7(b)).

In antioxidant-treated rats, the occurrence of T22-positive cells in both the GrDG and PoDG at seven days after blast was significantly reduced (all *p* < 0.01 or 0.001, Figure 7), although the prevalence of oligomeric Tau in the PoDG was not reduced to the baseline levels observed in uninjured animals (*p* < 0.05, Figure 7(a)). Along the breadth of the infrapyramidal blade of the GrDG, combinatorial antioxidant treatment reduced the number of T22-positive granular cells by more than 54% at this time point after blast (*p* < 0.001, Figure 7(b)). While we also noted accumulation of oligomeric Tau in the GrDG at both earlier (24 hours) and later (21 days) time points after blast injury (*p* < 0.01 or 0.001, Figure 7(b)), the treatment effects of antioxidants on T22 immunoreactivity were not significantly different from untreated controls at these time points (all *p* > 0.05, Figure 7(b)).

### 3.4. Identification of Pathologic Tau-Positive Cells in the Polymorphic Layer of the Dentate Gyrus

The blast-induced accumulation of hyperphosphorylated and oligomeric Tau in the PoDG of the hippocampus occurred primarily in cells that were morphologically distinct from the oligodendrocytes in which somatic Tau-1 immunoreactivity was primarily observed (Figures 1, 4, and 6). To more precisely identify this population of blast-sensitive cell types, immunofluorescence evaluations using antibodies against either calretinin or glutamate receptor 2/3 (GluR2/3) were conducted among Tau-1, AT8-, and T22-positive cells in the hippocampus. Calretinin is a specific marker for nonpyramidal *γ*-aminobutyric acid- (GABA-) ergic neurons within the adult hippocampus [39, 40], while GluR2/3 is a marker for mossy cells, the major excitatory neurons in the hilus of the dentate gyrus [41, 42].

**Figure 8:**
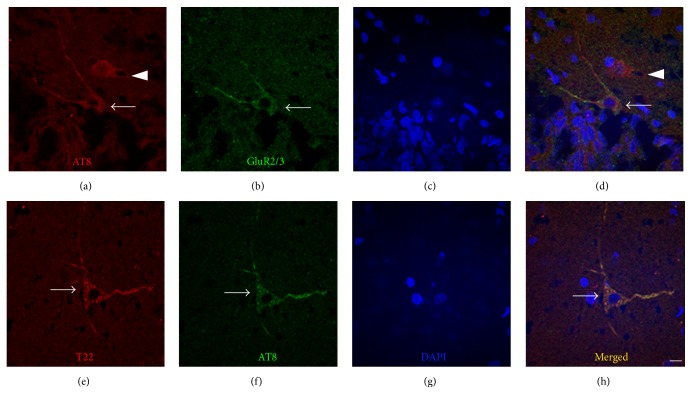
Tau accumulates in hippocampal cells in response to bTBI seven days after blast exposure. Representative images of AT8 and GluR2/3 ((a)–(d)), oligomeric Tau (T22) and AT8 ((e)–(h)) fluorescent immunolabeling in the PoDG of the hippocampus. In untreated rats, virtually AT8-positive cells in the hippocampus exhibited colabeling with the GluR2/3 antibody, identifying them as Mossy cells (arrow in (d)) while some AT8 positive cells were not colabeled with the GluR2/3 antibody (arrowhead in (d)). T22-positive cells in the hippocampus exhibited colabeling with the AT8 antibody (arrows in (e) and (h)). Scale bar in (h) = 10 *μ*m for (a)–(h).

A majority of the AT8-positive cells in blast-exposed animals exhibited colabeling with the GluR2/3 antibody (Figures 8(a)–8(d)). These results suggest that hilar mossy cells have a propensity to hyperphosphorylate Tau in response to our model of bTBI. A few AT8-positive cells were also colabeled with the calretinin antibody (data not shown), indicating that some inhibitory interneurons are also susceptible to this blast-induced hyperphosphorylation response. However, most AT8-positive cells did not exhibit calretinin immunolabeling (data not shown), consistent with our observation that mossy cells were the primary sites of Tau hyperphosphorylation in response to blast. In support of our initial immunohistochemical evaluations, none of the somatic Tau-1-positive cells that accumulated in the PoDG in response to blast colabeled with either calretinin or GluR2/3, validating their classification as morphologically distinct oligodendrocytes (data not shown).

To identify the cell types in which pathologic Tau oligomers accumulated within the PoDG in response to bTBI and the potential for their colocalization with hyperphosphorylated Tau, we performed dual labeling immunofluorescence with the T22 and AT8 antibodies. This analysis confirmed the blast-induced accumulation of oligomeric Tau in the PoDG of the hippocampus (Figures 8(e)–8(h)) and also revealed that both hyperphosphorylation and aggregation of Tau occurred in the same cells within the PoDG of the hippocampus (arrows in Figures 8(e), 8(f), and 8(h)), as virtually all T22-positive cells were colabeled with the AT8 antibody. These results indicate that mossy cells are the primary sites in the PoDG for both pathologic hyperphosphorylation and oligomerization of Tau in response to blast.

## 4. Discussion

In the present study, we evaluated the effects of a combinatorial antioxidant treatment regimen on the histopathology of the microtubule-associated protein, Tau, in the brains of nontransgenic rats following three successive, 14 psi blast overpressure exposures. In untreated brains, we discerned that the vast majority of atypical Tau accumulation in response to blast occurred in the hippocampus and that this effect could be significantly attenuated by therapeutic intervention with the antioxidants,* N*-acetylcysteine and HPN-07. Consistent with these observations, blast-induced neurotraumas have been shown to induce metabolic cascades that result in both diffuse axonal injury and neuron loss in the hippocampus in the acute period following blast exposures and progressive losses thereafter [4, 18, 43] and that these blast-induced insults have detrimental neurobehavioral consequences (i.e., deficits in spatial learning and memory) [44, 45]. As oxidative stress has been shown to be both a primary component of this maladaptive response in the hippocampus and a predictive factor for the extent of conformational changes that destabilize Tau [7, 18–23], the therapeutic efficacy of our combinatorial antioxidant treatment regimen represents a promising avenue for further clinical development in treating bTBI-related injuries.

### 4.1. Effects of Blast Exposure and Antioxidant Treatment on the Adverse Accumulation of Normal Tau in the Hippocampus

In our model of successive blast overpressure exposure, normal Tau markedly accumulated in the somata of oligodendrocytes. Oligodendrocytes are myelin-forming glial cells of the CNS, which are highly sensitive to oxidative stress, excitotoxicity, deprivation of trophic factors, and activation of apoptotic pathways [46]. While normally abundant in neurons of the central nervous system, Tau is typically expressed at very low levels in oligodendrocytes [47]. As such, prevalent Tau immunoreactivity has been found to be a sensitive marker for oligodendrocyte injury in models of focal cerebral ischemia and spinal cord injury in rats [28–30, 32] and in instances of brain injury in human patients [31]. In our blast model, the accumulation of Tau-positive oligodendrocytes occurred in a delayed (i.e., not obvious at 24 hours after blast) yet persistent (i.e., evident from 7–21 days after blast) fashion in the polymorphic layer of the dentate gyrus, suggestive of a progressive pathophysiological response. However, in blast-exposed rats that were subsequently treated with the combinatorial antioxidant treatment (NAC + HPN-07), the levels of somatic Tau in hippocampal oligodendrocytes were indistinguishable from naïve (i.e., sham blast exposed) controls at all time points. These results indicate that early antioxidant intervention is capable of interrupting the ongoing pathological events that culminate in the adverse accumulation of Tau in these cells. Thus, preserving the functional integrity of oligodendrocytes by short-circuiting blast-induced oxidative stress and aberrant Tau activity with combinatorial antioxidant intervention could have profound benefit in promoting both the short-term and long-term viability of neurotransmission in the hippocampus.

Previous studies have shown that somatic Tau accumulation can be detected in oligodendrocytes as early as a few hours after a TBI in both rodents and humans [29, 31, 46, 48–50]. The delayed accumulation of somatic Tau in hippocampal oligodendrocytes in our model of bTBI may be attributable to the relatively mild blast intensity to which the animals were exposed, resulting in chronic rather than acute mechanical disruption. A recent study in mice revealed a biphasic pattern of normal Tau accumulation, in which normal Tau levels in the brain initially declined at six hours after injury and then increased at 24 hours in response to three closely coupled higher-intensity blast exposures (21 psi, separated by 1 and 30 min) [35]. Other studies have shown that Tau levels increase rapidly in serum and cerebrospinal fluid or the brain extracellular space after traumatic brain injury, and the relative levels of extracellular Tau may be a reflection of the severity and extent of the brain injury and can be correlated with clinical outcome [51–54]. Nonetheless, the sustained elevation of somatic Tau levels in hippocampal oligodendrocytes observed in our model of bTBI is consistent with previous studies of Tau accumulation in the brains of mice in response to mild blast exposures (11.18 or 16 psi) [11, 19], albeit in a delayed fashion, indicative of ongoing injury. This type of somatodendritic translocation has been shown to destabilize microtubules and impair axonal transport of mitochondria between the nucleus and synapse, leading to severe energy deprivation, free radical generation, and, ultimately, synaptic failure [55–57]. Functional impairment or loss of myelin-producing oligodendrocytes in the hippocampus in response to blast could lead to demyelination and, thus, destabilization of neuronal axons, with significant implications for impulse dynamics and neural plasticity [58].

### 4.2. Pathological Variants of Tau Accumulate in Hilar Mossy Cells in the Hippocampus following Mild bTBI

Phosphorylation of Tau is regulated by a host of serine/threonine kinases, including glycogen synthase kinase-3*β* and cyclin-dependent kinase 5 [59]. In uninjured brains, Tau is primarily localized to the axons of mature neurons [60]. Under conditions of pathologic stress, Tau is susceptible to hyperphosphorylation, which can result in dissociation and mislocalization of Tau from axonal microtubules and subsequent oligomerization of Tau monomers, thus promoting disassembly of the neuronal cytoskeleton [61, 62]. Redistribution of hyperphosphorylated Tau to the somatodendritic compartment is considered a key pathological indicator during the early development of tauopathic disorders [63, 64].

In response to mild successive blast overpressures, we observed a marked increase in the number of cells immunopositive for hyperphosphorylated and oligomeric variants of Tau in the rat hippocampus, indicative of Tau dysfunction in this region of the brain. Using differential immunofluorescence, we were able to discern that both of these pathological Tau variants preferentially accumulated within the somata of hilar mossy cells within the polymorphic layer of the dentate gyrus. Mossy cells are the major excitatory neurons in the PoDG, where they participate in recurrent excitatory circuits with granule cells and play an important role in normal signal processing associated with learning and memory [41, 42, 65, 66]. Extensive loss of hilar mossy cells can cause granule cell hyperexcitability, a maladaptive TBI response which has been documented previously in the dentate gyrus in rats and which may contribute to the development of posttraumatic epilepsy [67–69]. Like oligodendrocytes, mossy cells are particularly vulnerable to excitotoxic insults or neurotraumatic events, including hypoxia/ischemia and fluid-percussive brain injuries [66, 68, 70, 71]. The present study indicates that mossy cells are also preferentially vulnerable to the accumulation of neurotoxic variants of Tau in response to mild repetitive blast exposure.

Historically, NFTs, primarily composed of hyperphosphorylated Tau, have been considered the main histopathological hallmarks for neurodegenerative tauopathies, such as Alzheimer's and Parkinson's diseases [72]. It has recently been suggested that the development of neurofibrillary tangles may also serve as a diagnostic neuropathological feature of individuals exposed to acute blast-induced brain traumas [11, 73, 74]. However, neurodegeneration and decreased cognition often precede the formation of NFTs among tauopathies [75–78]. Recent studies indicate that prefilamentous Tau oligomers are capable of inducing synaptic dysfunction and neuronal cell death [79, 80]. Etiologically, small Tau oligomers often portend the development of future NFTs and are sufficient for inducing neurotoxicity* in vivo* [37].

### 4.3. Treatment Effects on Tau Expression in the Hippocampus

A positive correlation exists between oxidative stress and the induction of aberrant patterns of Tau functional regulation [57]. In transgenic murine models of Alzheimer's disease, for instance, animals hemizygous for superoxide dismutase 2 exhibit a marked increase in Tau hyperphosphorylation in response to mitochondrial oxidative stress, and daily injections with a manganese-containing catalytic antioxidant were able to ameliorate this potentially damaging effect [81]. Moreover, it has been found that phosphorylated Tau protein is induced to aggregate after reaction with 4-hydroxy-2-nonenal (4-HNE), a lipid peroxidation byproduct formed during oxidative stress [16, 82]. These findings suggest that oxidative stress conditions may play a key role in the etiopathology of progressive tauopathies. Consistent with this hypothesis, oxidative stress has been observed as an early pathological indicator in brains exposed to blast overpressures, and the hippocampus has been documented as a primary site for this response [7, 18–22]. These findings may explain why persistent somatodendritic accumulation of dysfunctional Tau is commonly observed in the hippocampus in response to blast [11, 19, 36, 44]. We have previously demonstrated that 4-HNE accumulates to high levels in the hippocampus within the first 24 hours following blast exposure in our model of mild bTBI [7]. We have also shown that amyloid *β* precursor protein (APP) accumulates to high levels in the hippocampus during this acute time period following blast exposure [7]. In light of the fact that dysregulation of APP function has been shown to potentiate Tau hyperphosphorylation in response to mitochondrial stress [77], the early accumulation of reactive oxygen species (e.g., 4-HNE) and APP in the hippocampus may induce or contribute to the subsequent pathological changes in Tau protein identified herein.

We had previously demonstrated that antioxidant intervention protects against progressive injury to the central auditory pathway in rats following exposure to blast overpressures [7, 24]. In the present study, these analyses have been extended to evaluate the therapeutic effects of posttraumatic intraperitoneal injection of the antioxidants HPN-07 and NAC on the histopathology of Tau in the hippocampus in this rodent model of bTBI. Treatment effects were observed in the hippocampus among all histopathological manifestations of Tau proteins examined, including somatodendritic Tau, hyperphosphorylated Tau, and oligomeric Tau. These findings suggest that the antioxidant treatment regimen employed in the present study has the potential to attenuate the progressive mislocalization, phosphorylation, and oligomerization of Tau with the aim of providing neural protection to the brain. In our previous studies, we were able to demonstrate that HPN-07 and NAC treatment could significantly reduce early blast-induced 4-HNE production and APP accumulation in the hippocampus and oppose associated long-term progressive injury to hippocampal neurons [7]. The ability of HPN-07 and NAC to ameliorate the delayed, yet persistent, blast-induced tau dysfunction described herein may be a direct reflection of the therapeutic efficacy of these antioxidants to interrupt the chain of events that culminate in the long-term loss of neurons documented in untreated (i.e., blast alone) controls [7].

Consistent with these observations, HPN-07 has been shown to inhibit the production of reactive oxygen species and reduce cognitive dysfunction when administered to animals following percussion-induced traumatic brain injury or in experimental models of stroke [83–86]. Moreover, an *α*-phenyl-tert-butyl-nitrone (PBN, a structural ortholog of HPN-07) pretreatment regimen has also been shown to reduce the number of Tau-positive oligodendrocytes by 55% in the brains of rats subjected to ischemic brain injury [49]. NAC treatment provides generalized neuroprotection* in vitro* and* in vivo* by inhibiting toxin or trauma-induced apoptosis and glutamate-excitotoxicity and enhancing mitochondrial protection [87–90]. Administration of NAC after focal cerebral ischemia protects brains from free radical injury, apoptosis, and inflammation in rat models of experimental stroke [91, 92].

In our model of bTBI, NAC and HPN-07 likely cooperate to oppose Tau dysregulation through both their antioxidant and anti-inflammatory properties, consistent with the strong links between oxidative stress, inflammation, and Tau hyperphosphorylation [57, 93–96]. However, it is probable that these two therapeutic agents employ both overlapping and nonoverlapping mechanisms to mediate this protective function. For instance, of the two, NAC uniquely serves as a substrate for replenishing the endogenous stores of glutathione in the brain, the levels of which often become overwhelmed following an acute traumatic brain injury [97]. Consistent with this rationale,* in vitro* studies with M17 neuroblastoma cells have demonstrated that loss of glutathione through inhibition of glutathione synthesis promotes oxidizing conditions that lead to hyperphosphorylation of Tau [93]. It should be noted, however, that, under conditions in which endogenous antioxidant activity is diminished, accumulated cysteine has a high propensity to react with NAC, resulting in the acceleration of cysteine autoxidation and the undesirable production of hydrogen peroxide and other free radicals [98, 99]. Under these circumstances, the prooxidant potential of NAC may be adversely weighed against its therapeutic antioxidant properties. Coadministration of HPN-07 may, thus, act as a sink for these neurotoxic byproducts, potentiating the therapeutic benefit of NAC under oxidative stress conditions by reducing its prooxidant risk factor.

In addition to their ability to scavenge free radicals, the known pharmacological effects of nitrones (e.g., HPN-07) are primarily anti-inflammatory [100]. Many neurodegenerative diseases involve a degree of neuroinflammation in which inappropriate damage to healthy bystander tissue is induced by proinflammatory cytokines and neurotoxins produced by activated microglia and astrocytes [101]. In the case of tauopathies, microglial activation has been shown to precede and drive dysregulation of Tau function in the hippocampus [102, 103]. The parent compound of HPN-07, PBN, is known to limit microglial activation and inhibit the proinflammatory activity of p38 and NF-*κ*B induced by acute neurotoxic insults [104]. More specifically, NXY-059 (aka HPN-07) treatment was documented to significantly reduce the neutrophil infiltrate observed 48 hours after hemorrhage in the vicinity of hematomas in a rat model of hemorrhagic stroke [105]. Thus, the combined antioxidant and anti-inflammatory potentials of HPN-07 and NAC are seemingly well-suited for therapeutically opposing hippocampal Tau dysfunction.

Other antioxidants have shown positive treatment effects for reducing Tau hyperphosphorylation and improving spatial learning and memory in animal models of Alzheimer's disease [106–108]. More specifically, an amide derivative of NAC has recently been shown to significantly improve cognitive function following TBI [109]. Thus, the ability of HPN-07 and NAC combinatorial treatment to elicit protection among unique blast-induced manifestations of aberrant Tau function in two distinct, yet functionally critical, hippocampal cell populations lends support for its therapeutic utility in treating blast injuries. While the apparent antioxidant-mediated protection of oligodendrocytes was still evident at 21 days after injury, the long-term treatment effects observed for hyperphosphorylated Tau or oligomeric Tau (T22) in hilar mossy cells was less pronounced at 21 days after injury, suggesting that a longer course of antioxidant treatment may be needed for long-term protection of this cell population. Future studies will be aimed at addressing this issue and testing the therapeutic efficacy of this combinatorial antioxidant treatment on cognitive functional recovery following bTBI.

## Abbreviations

bTBI: Blast-induced traumatic brain injury
CTE: Chronic traumatic encephalopathy
GABA: *γ*-Aminobutyric acid
GluR2/3: Glutamate receptor 2/3
GrDG: Granular layer of the dentate gyrus
4-HNE: 4-Hydroxy-2-nonenal
HPN-07: 2,4-Disulfonyl *α*-phenyl tertiary butyl nitrone
MoDG: Molecular layer of the dentate gyrus
NAC: N-Acetylcysteine
NFT: Neurofibrillary tangle
OSPA: Oligodendrocyte specific protein antibody
PHF: Paired helical filament
PoDG: Polymorphic layer of the dentate gyrus
TBI: Traumatic brain injury.

## References

[1] Baalman K. L., Cotton R. J., Rasband S. N., Rasband M. N. (2013). Blast wave exposure impairs memory and decreases axon initial segment length. *Journal of Neurotrauma*.

[2] Garman R. H., Jenkins L. W., Switzer R. C. (2011). Blast exposure in rats with body shielding is characterized primarily by diffuse axonal injury. *Journal of Neurotrauma*.

[3] Kuehn R., Simard P. F., Driscoll I. (2011). Rodent model of direct cranial blast injury. *Journal of Neurotrauma*.

[4] Lu J., Ng K. C., Ling G. (2012). Effect of blast exposure on the brain structure and cognition in *Macaca fascicularis*. *Journal of Neurotrauma*.

[5] Magnuson J., Leonessa F., Ling G. S. F. (2012). Neuropathology of explosive blast traumatic brain injury. *Current Neurology and Neuroscience Reports*.

[6] de Lanerolle N. C., Bandak F., Kang D. (2011). Characteristics of an explosive blast-induced brain injury in an experimental model. *Journal of Neuropathology and Experimental Neurology*.

[7] Du X., Ewert D. L., Cheng W. (2013). Effects of antioxidant treatment on blast-induced brain injury. *PLoS ONE*.

[8] Folmer R. L., Billings C. J., Diedesch-Rouse A. C., Gallun F. J., Lew H. L. (2011). Electrophysiological assessments of cognition and sensory processing in TBI: applications for diagnosis, prognosis and rehabilitation. *International Journal of Psychophysiology*.

[9] Tang-Schomer M. D., Johnson V. E., Baas P. W., Stewart W., Smith D. H. (2012). Partial interruption of axonal transport due to microtubule breakage accounts for the formation of periodic varicosities after traumatic axonal injury. *Experimental Neurology*.

[10] Walker K. R., Tesco G. (2013). Molecular mechanisms of cognitive dysfunction following traumatic brain injury. *Frontiers in Aging Neuroscience*.

[11] Goldstein L. E., Fisher A. M., Tagge C. A. (2012). Chronic traumatic encephalopathy in blast-exposed military veterans and a blast neurotrauma mouse model. *Science Translational Medicine*.

[12] Johnson G. V. W., Stoothoff W. H. (2004). Tau phosphorylation in neuronal cell function and dysfunction. *Journal of Cell Science*.

[13] Clavaguera F., Lavenir I., Falcon B., Frank S., Goedert M., Tolnay M. (2013). ‘Prion-like’ templated misfolding in tauopathies. *Brain Pathology*.

[14] Guo J. L., Lee V. M.-Y. (2011). Seeding of normal tau by pathological tau conformers drives pathogenesis of Alzheimer-like tangles. *The Journal of Biological Chemistry*.

[15] Wolfe M. S. (2012). The role of tau in neurodegenerative diseases and its potential as a therapeutic target. *Scientifica*.

[16] Pérez M., Cuadros R., Smith M. A., Perry G., Avila J. (2000). Phosphorylated, but not native, tau protein assembles following reaction with the lipid peroxidation product, 4-hydroxy-2-nonenal. *FEBS Letters*.

[17] Abdul-Muneer P. M., Schuetz H., Wang F. (2013). Induction of oxidative and nitrosative damage leads to cerebrovascular inflammation in an animal model of mild traumatic brain injury induced by primary blast. *Free Radical Biology and Medicine*.

[18] Cernak I., Wang Z., Jiang J., Bian X., Savic J. (2001). Ultrastructural and functional characteristics of blast injury-induced neurotrauma. *Journal of Trauma—Injury, Infection and Critical Care*.

[19] Huber B. R., Meabon J. S., Martin T. J. (2013). Blast exposure causes early and persistent aberrant phospho- and cleaved-tau expression in a murine model of mild blast-induced traumatic brain injury. *Journal of Alzheimer's Disease*.

[20] Kochanek P. M., Dixon C. E., Shellington D. K. (2013). Screening of biochemical and molecular mechanisms of secondary injury and repair in the brain after experimental blast-induced traumatic brain injury in rats. *Journal of Neurotrauma*.

[21] Readnower R. D., Chavko M., Adeeb S. (2010). Increase in blood-brain barrier permeability, oxidative stress, and activated microglia in a rat model of blast-induced traumatic brain injury. *Journal of Neuroscience Research*.

[22] Sajja V. S. S. S., Ereifej E. S., VandeVord P. J. (2014). Hippocampal vulnerability and subacute response following varied blast magnitudes. *Neuroscience Letters*.

[23] Valiyaveettil M., Alamneh Y., Miller S.-A. (2012). Preliminary studies on differential expression of auditory functional genes in the brain after repeated blast exposures. *Journal of Rehabilitation Research and Development*.

[24] Ewert D. L., Lu J., Li W., Du X., Floyd R. A., Kopke R. D. (2012). Antioxidant treatment reduces blast-induced cochlear damage and hearing loss. *Hearing Research*.

[25] Du X., Chen K., Choi C.-H. (2012). Selective degeneration of synapses in the dorsal cochlear nucleus of chinchilla following acoustic trauma and effects of antioxidant treatment. *Hearing Research*.

[26] Lu J., Li W., Du X. (2014). Antioxidants reduce cellular and functional changes induced by intense noise in the inner ear and cochlear nucleus. *Journal of the Association for Research in Otolaryngology*.

[27] Binder L. I., Frankfurter A., Rebhun L. I. (1985). The distribution of tau in the mammalian central nervous system. *The Journal of Cell Biology*.

[28] Dewar D., Dawson D. (1995). Tau protein is altered by focal cerebral ischaemia in the rat: an immunohistochemical and immunoblotting study. *Brain Research*.

[29] Gresle M. M., Jarrott B., Jones N. M., Callaway J. K. (2006). Injury to axons and oligodendrocytes following endothelin-1-induced middle cerebral artery occlusion in conscious rats. *Brain Research*.

[30] Imai H., Masayasu H., Dewar D., Graham D. I., Macrae I. M. (2001). Ebselen protects both gray and white matter in a rodent model of focal cerebral ischemia. *Stroke*.

[31] Irving E. A., Nicoll J., Graham D. I., Dewar D. (1996). Increased tau immunoreactivity in oligodendrocytes following human stroke and head injury. *Neuroscience Letters*.

[32] McCracken E., Fowler J. H., Dewar D., Morrison S., McCulloch J. (2002). Grey matter and white matter ischemic damage is reduced by the competitive AMPA receptor antagonist, SPD 502. *Journal of Cerebral Blood Flow and Metabolism*.

[33] Xiao F., Fei M., Cheng C. (2008). Spatiotemporal patterns of SSeCKS expression after rat spinal cord injury. *Neurochemical Research*.

[34] Stoothoff W. H., Johnson G. V. W. (2005). Tau phosphorylation: physiological and pathological consequences. *Biochimica et Biophysica Acta*.

[35] Arun P., Abu-Taleb R., Oguntayo S. (2013). Distinct patterns of expression of traumatic brain injury biomarkers after blast exposure: role of compromised cell membrane integrity. *Neuroscience Letters*.

[36] Hawkins B. E., Krishnamurthy S., Castillo-Carranza D. L. (2013). Rapid accumulation of endogenous tau oligomers in a rat model of traumatic brain injury: possible link between traumatic brain injury and sporadic tauopathies. *The Journal of Biological Chemistry*.

[37] Lasagna-Reeves C. A., Castillo-Carranza D. L., Sengupta U., Clos A. L., Jackson G. R., Kayed R. (2011). Tau oligomers impair memory and induce synaptic and mitochondrial dysfunction in wild-type mice. *Molecular Neurodegeneration*.

[38] Wu J. W., Herman M., Liu L. (2013). Small misfolded tau species are internalized via bulk endocytosis and anterogradely and retrogradely transported in neurons. *The Journal of Biological Chemistry*.

[39] Gulyás A. I., Miettinen R., Jacobowitz D. M., Freund T. F. (1992). Calretinin is present in non-pyramidal cells of the rat hippocampus—I. A new type of neuron specifically associated with the mossy fibre system. *Neuroscience*.

[40] Miettinen R., Gulyás A. I., Baimbridge K. G., Jacobowitz D. M., Freund T. F. (1992). Calretinin is present in non-pyramidal cells of the rat hippocampus—II. Co-existence with other calcium binding proteins and gaba. *Neuroscience*.

[41] Duffy A. M., Schaner M. J., Chin J., Scharfman H. E. (2013). Expression of c-fos in hilar mossy cells of the dentate gyrus *in vivo*. *Hippocampus*.

[42] Ratzliff A. D. H., Howard A. L., Santhakumar V., Osapay I., Soltesz I. (2004). Rapid deletion of mossy cells does not result in a hyperexcitable dentate gyrus: implications for epileptogenesis. *The Journal of Neuroscience*.

[43] Sajja V. S. S. S., Galloway M. P., Ghoddoussi F. (2012). Blast-induced neurotrauma leads to neurochemical changes and neuronal degeneration in the rat hippocampus. *NMR in Biomedicine*.

[44] Kovesdi E., Gyorgy A. B., Kwon S.-K. C. (2011). The effect of enriched environment on the outcome of traumatic brain injury; a behavioral, proteomics, and histological study. *Frontiers in Neuroscience*.

[45] Kwon S.-K. C., Kovesdi E., Gyorgy A. B. (2011). Stress and traumatic brain injury: a behavioral, proteomics, and histological study. *Frontiers in Neurology*.

[46] Dewar D., Underhill S. M., Goldberg M. P. (2003). Oligodendrocytes and ischemic brain injury. *Journal of Cerebral Blood Flow and Metabolism*.

[47] Shin R.-W., Iwaki T., Kitamoto T., Tateishi J. (1991). Hydrated autoclave pretreatment enhances tau immunoreactivity in formalin-fixed normal and Alzheimer's disease brain tissues. *Laboratory Investigation*.

[48] Hughes P. M., Anthony D. C., Ruddin M. (2003). Focal lesions in the rat central nervous system induced by endothelin-1. *Journal of Neuropathology and Experimental Neurology*.

[49] Irving E. A., Yatsushiro K., McCulloch J., Dewar D. (1997). Rapid alteration of tau in oligodendrocytes after focal ischemic injury in the rat: involvement of free radicals. *Journal of Cerebral Blood Flow and Metabolism*.

[50] Smith C., Graham D. I., Murray L. S., Nicoll J. A. R. (2003). Tau immunohistochemistry in acute brain injury. *Neuropathology and Applied Neurobiology*.

[51] Franz G., Beer R., Kampfl A. (2003). Amyloid beta 1-42 and tau in cerebrospinal fluid after severe traumatic brain injury. *Neurology*.

[52] Liliang P.-C., Liang C.-L., Lu K. (2010). Relationship between injury severity and serum tau protein levels in traumatic brain injured rats. *Resuscitation*.

[53] Magnoni S., Esparza T. J., Conte V. (2012). Tau elevations in the brain extracellular space correlate with reduced amyloid-*β* levels and predict adverse clinical outcomes after severe traumatic brain injury. *Brain*.

[54] Zemlan F. P., Rosenberg W. S., Luebbe P. A. (1999). Quantification of axonal damage in traumatic brain injury: affinity purification and characterization of cerebrospinal fluid tau proteins. *Journal of Neurochemistry*.

[55] Kopeikina K. J., Carlson G. A., Pitstick R. (2011). Tau accumulation causes mitochondrial distribution deficits in neurons in a mouse model of tauopathy and in human Alzheimer's disease brain. *The American Journal of Pathology*.

[56] Rapoport S. I. (2003). Coupled reductions in brain oxidative phosphorylation and synaptic function can be quantified and staged in the course of Alzheimer disease. *Neurotoxicity Research*.

[57] Mondragón-Rodríguez S., Perry G., Zhu X., Moreira P. I., Acevedo-Aquino M. C., Williams S. (2013). Phosphorylation of tau protein as the link between oxidative stress, mitochondrial dysfunction, and connectivity failure: implications for Alzheimer's disease. *Oxidative Medicine and Cellular Longevity*.

[58] Yeung M. S. Y., Zdunek S., Bergmann O. (2014). Dynamics of oligodendrocyte generation and myelination in the human brain. *Cell*.

[59] Wang J.-Z., Xia Y.-Y., Grundke-Iqbal I., Iqbal K. (2013). Abnormal hyperphosphorylation of tau: sites, regulation, and molecular mechanism of neurofibrillary degeneration. *Journal of Alzheimer's Disease*.

[60] Hirokawa N., Funakoshi T., Sato-Harada R., Kanai Y. (1996). Selective stabilization of tau in axons and microtubule-associated protein 2C in cell bodies and dendrites contributes to polarized localization of cytoskeletal proteins in mature neurons. *The Journal of Cell Biology*.

[61] Noble W., Hanger D. P., Miller C. C. J., Lovestone S. (2013). The importance of tau phosphorylation for neurodegenerative diseases. *Frontiers in Neurology*.

[62] Taniguchi T., Kawamata T., Mukai H. (2001). Phosphorylation of tau is regulated by PKN. *The Journal of Biological Chemistry*.

[63] Andorfer C., Kress Y., Espinoza M. (2003). Hyperphosphorylation and aggregation of tau in mice expressing normal human tau isoforms. *Journal of Neurochemistry*.

[64] Braak H., Thal D. R., Ghebremedhin E., Del Tredici K. (2011). Stages of the pathologic process in Alzheimer disease: age categories from 1 to 100 years. *Journal of Neuropathology and Experimental Neurology*.

[65] Lisman J. E. (1999). Relating hippocampal circuitry to function: recall of memory sequences by reciprocal dentate-CA3 interactions. *Neuron*.

[66] Scharfman H. E., Myers C. E. (2013). Hilar mossy cells of the dentate gyrus: a historical perspective. *Frontiers in Neural Circuits*.

[67] Golarai G., Greenwood A. C., Feeney D. M., Connor J. A. (2001). Physiological and structural evidence for hippocampal involvement in persistent seizure susceptibility after traumatic brain injury. *The Journal of Neuroscience*.

[68] Santhakumar V., Bender R., Frotscher M. (2000). Granule cell hyperexcitability in the early post-traumatic rat dentate gyrus: the ‘irritable mossy cell’ hypothesis. *The Journal of Physiology*.

[69] Sloviter R. S. (1994). The functional organization of the hippocampal dentate gyrus and its relevance to the pathogenesis of temporal lobe epilepsy. *Annals of Neurology*.

[70] Hsu M., Buzsáki G. (1993). Vulnerability of mossy fiber targets in the rat hippocampus to forebrain ischemia. *The Journal of Neuroscience*.

[71] Lowenstein D. H., Thomas M. J., Smith D. H., McIntosh T. K. (1992). Selective vulnerability of dentate hilar neurons following traumatic brain injury: a potential mechanistic link between head trauma and disorders of the hippocampus. *The Journal of Neuroscience*.

[72] Gerson J. E., Kayed R. (2013). Formation and propagation of tau oligomeric seeds. *Frontiers in Neurology*.

[73] Lucke-Wold B. P., Turner R. C., Logsdon A. F., Bailes J. E., Huber J. D., Rosen C. L. (2014). Linking traumatic brain injury to chronic traumatic encephalopathy: identification of potential mechanisms leading to neurofibrillary tangle development. *Journal of Neurotrauma*.

[74] Miller G. (2012). Neuropathology. Blast injuries linked to neurodegeneration in veterans. *Science*.

[75] Gómez-Isla T., Hollister R., West H. (1997). Neuronal loss correlates with but exceeds neurofibrillary tangles in Alzheimer's disease. *Annals of Neurology*.

[76] Haroutunian V., Davies P., Vianna C., Buxbaum J. D., Purohit D. P. (2007). Tau protein abnormalities associated with the progression of Alzheimer disease type dementia. *Neurobiology of Aging*.

[77] Kril J. J., Patel S., Harding A. J., Halliday G. M. (2002). Neuron loss from the hippocampus of Alzheimer's disease exceeds extracellular neurofibrillary tangle formation. *Acta Neuropathologica*.

[78] Morsch R., Simon W., Coleman P. D. (1999). Neurons may live for decades with neurofibrillary tangles. *Journal of Neuropathology and Experimental Neurology*.

[79] Berger Z., Roder H., Hanna A. (2007). Accumulation of pathological tau species and memory loss in a conditional model of tauopathy. *The Journal of Neuroscience*.

[80] Cowan C. M., Quraishe S., Mudher A. (2012). What is the pathological significance of tau oligomers?. *Biochemical Society Transactions*.

[81] Melov S., Adlard P. A., Morten K. (2007). Mitochondrial oxidative stress causes hyperphosphorylation of tau. *PLoS ONE*.

[82] Takeda A., Smith M. A., Avilá J. (2000). In Alzheimer's disease, heme oxygenase is coincident with Alz50, an epitope of *τ* induced by 4-hydroxy-2-nonenal modification. *Journal of Neurochemistry*.

[83] Clausen F., Marklund N., Lewén A., Hillered L. (2008). The nitrone free radical scavenger NXY-059 is neuroprotective when administered after traumatic brain injury in the rat. *Journal of Neurotrauma*.

[84] Han M., He Q. P., Yong G., Siesjö B. K., Li P. A. (2003). NXY-059, a nitrone with free radical trapping properties inhibits release of cytochrome c after focal cerebral ischemia. *Cellular and Molecular Biology*.

[85] Marshall J. W. B., Duffin K. J., Green A. R., Ridley R. M. (2001). NXY-059, a free radical—trapping agent, substantially lessens the functional disability resulting from cerebral ischemia in a primate species. *Stroke*.

[86] Zhao Z., Cheng M., Maples K. R., Ma J. Y., Buchan A. M. (2001). NXY-059, a novel free radical trapping compound, reduces cortical infarction after permanent focal cerebral ischemia in the rat. *Brain Research*.

[87] Hart A. M., Terenghi G., Kellerth J.-O., Wiberg M. (2004). Sensory neuroprotection, mitochondrial preservation, and therapeutic potential of *N*-acetyl-cysteine after nerve injury. *Neuroscience*.

[88] Papa L., Gomes E., Rockwell P. (2007). Reactive oxygen species induced by proteasome inhibition in neuronal cells mediate mitochondrial dysfunction and a caspase-independent cell death. *Apoptosis: An International; Journal on Programmed Cell Death*.

[89] Stephenson A. P., Schneider J. A., Nelson B. C. (2013). Manganese-induced oxidative DNA damage in neuronal SH-SY5Y cells: attenuation of thymine base lesions by glutathione and *N*-acetylcysteine. *Toxicology Letters*.

[90] Floyd R. A., Kopke R. D., Choi C.-H., Foster S. B., Doblas S., Towner R. A. (2008). Nitrones as therapeutics. *Free Radical Biology and Medicine*.

[91] Khan M., Sekhon B., Jatana M. (2004). Administration of N-acetylcysteine after focal cerebral ischemia protects brain and reduces inflammation in a rat model of experimental stroke. *Journal of Neuroscience Research*.

[92] Sekhon B., Sekhon C., Khan M., Patel S. J., Singh I., Singh A. K. (2003). N-acetyl cysteine protects against injury in a rat model of focal cerebral ischemia. *Brain Research*.

[93] Su B., Wang X., Lee H.-G. (2010). Chronic oxidative stress causes increased tau phosphorylation in M17 neuroblastoma cells. *Neuroscience Letters*.

[94] Lee D. C., Rizer J., Selenica M.-L. B. (2010). LPS-induced inflammation exacerbates phospho-tau pathology in rTg4510 mice. *Journal of Neuroinflammation*.

[95] Sy M., Kitazawa M., Medeiros R. (2011). Inflammation induced by infection potentiates tau pathological features in transgenic mice. *The American Journal of Pathology*.

[96] Maphis N., Xu G., Kokiko-Cochran O. N. (2015). Loss of tau rescues inflammation-mediated neurodegeneration. *Frontiers in Neuroscience*.

[97] Dringen R. (2000). Metabolism and functions of glutathione in brain. *Progress in Neurobiology*.

[98] Sagara J.-I., Bannai S., Shikano N., Makino N. (2010). Conflicting effects of N-acetylcysteine on purified neurons derived from rat cortical culture. *NeuroReport*.

[99] Saez G., Thornalley P. J., Hill H. A. O., Hems R., Bannister J. V. (1982). The production of free radicals during the autoxidation of cysteine and their effect on isolated rat hepatocytes. *Biochimica et Biophysica Acta—General Subjects*.

[100] Floyd R. A., Neto H. C. C. F., Zimmerman G. A., Hensley K., Towner R. A. (2013). Nitrone-based therapeutics for neurodegenerative diseases: their use alone or in combination with lanthionines. *Free Radical Biology and Medicine*.

[101] Gao H.-M., Hong J.-S. (2008). Why neurodegenerative diseases are progressive: uncontrolled inflammation drives disease progression. *Trends in Immunology*.

[102] Yoshiyama Y., Higuchi M., Zhang B. (2007). Synapse loss and microglial activation precede tangles in a P301S tauopathy mouse model. *Neuron*.

[103] Maphis N., Xu G., Kokiko-Cochran O. N. (2015). Reactive microglia drive tau pathology and contribute to the spreading of pathological tau in the brain. *Brain*.

[104] Floyd R. A. (2006). Nitrones as therapeutics in age-related diseases. *Aging Cell*.

[105] Peeling J., Del Bigio M. R., Corbett D., Green A. R., Jackson D. M. (2001). Efficacy of disodium 4-[(tert-butylimino)methyl]benzene-1,3-disulfonate N-oxide (NXY-059), a free radical trapping agent, in a rat model of hemorrhagic stroke. *Neuropharmacology*.

[106] Prasanthi J. R. P., Dasari B., Marwarha G. (2010). Caffeine protects against oxidative stress and Alzheimer's disease-like pathology in rabbit hippocampus induced by cholesterol-enriched diet. *Free Radical Biology and Medicine*.

[107] Rajasekar N., Dwivedi S., Tota S. K. (2013). Neuroprotective effect of curcumin on okadaic acid induced memory impairment in mice. *European Journal of Pharmacology*.

[108] Shi T.-Y., Zhao D.-Q., Wang H.-B. (2013). A new chiral pyrrolyl *α*-nitronyl nitroxide radical attenuates *β*-amyloid deposition and rescues memory deficits in a mouse model of Alzheimer disease. *Neurotherapeutics: The Journal of the American Society for Experimental NeuroTherapeutics*.

[109] Pandya J. D., Readnower R. D., Patel S. P. (2014). N-acetylcysteine amide confers neuroprotection, improves bioenergetics and behavioral outcome following TBI. *Experimental Neurology*.

